# Nanocarbon-Edge-Anchored High-Density Pt Atoms for 3-nitrostyrene Hydrogenation: Strong Metal-Carbon Interaction

**DOI:** 10.1016/j.isci.2019.02.016

**Published:** 2019-02-20

**Authors:** Yang Lou, Honglu Wu, Jingyue Liu

**Affiliations:** 1 Department of Physics, Arizona State University, Tempe, AZ 85287, USA

**Keywords:** Chemical Reaction Engineering, Catalysis, Nanostructure

## Abstract

Strong metal-support interaction (SMSI) has been widely used to improve catalytic performance and to identify reaction mechanisms. We report that single Pt atoms anchored onto hollow nanocarbon (h-NC) edges possess strong metal-carbon interaction, which significantly modifies the catalytic behavior of the anchored Pt atoms for selective hydrogenation reactions. The strong Pt-C bonding not only stabilizes single Pt atoms but also modifies their electronic structure, tunes their adsorption properties, and enhances activation of reactants. The fabricated Pt_1_/h-NC single-atom catalysts (SACs) demonstrated excellent activity for hydrogenation of 3-nitrostyrene to 3-vinylaniline with a turnover number >31,000/h, 20 times higher than that of the best catalyst for such selective hydrogenation reactions reported in the literature. The strategy to strongly anchor Pt atoms by edge carbon atoms of h-NCs is general and can be extended to construct strongly anchored metal atoms, via SMSI, onto surfaces of various types of support materials to develop robust SACs.

## Highlights

• A strategy is proposed to anchor metal atoms to edge carbon atoms of graphene sheets
• Formation of Pt_1_-C bond tunes performance of Pt_1_/h-NC single-atom catalyst (SAC)
• Pt_1_/h-NC SAC is 20 times more active than the best catalyst for 3-nitrostyrene hydrogenation
• Electronic SMSI can be effectively used to tune catalytic properties of SACs

## Graphical Abstract

**Figure:**
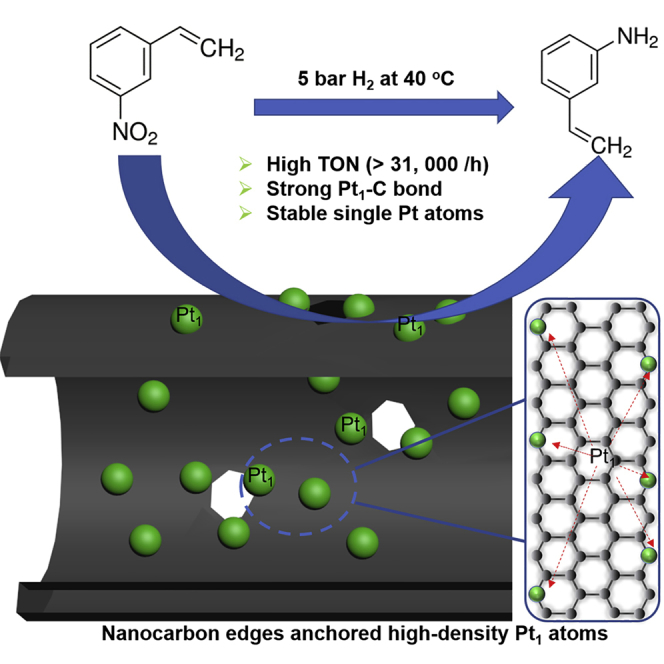

## Introduction

Supported metal catalysts with metal particles finely dispersed on high-surface-area support materials are vital for many industrially important catalytic reactions. The surface physicochemical properties of the supports play a crucial role in modifying the catalytic behaviors of supported metal species via the strong metal-support interaction (SMSI) (Lee et al., 2015, Tang et al., 2016a, Tang et al., 2016b; Vannice, 1979, Matsubu et al., 2017). The classical SMSI phenomenon was discovered on titania-supported Pt-group metals of which high temperature reduction significantly influenced the adsorption properties of small molecules such as H_2_ and CO (Tauster et al., 1978). It has been reported that pretreatment and reaction conditions induce the SMSI as well in oxides supported Au (for CO oxidation) (Liu et al., 2012) and Rh (for CO_2_ reduction by H_2_) catalysts (Matsubu et al., 2017), which are termed oxidative and adsorbate-mediated SMSI. Recent reports demonstrated that carbides (Dong et al., 2018), phosphates (Tang et al., 2016a, Tang et al., 2016b), and layered double hydroxides (Wang et al., 2017) supported Au catalysts exhibited the SMSI effects for water-gas shift, CO oxidation, and ethanol dehydrogenation reactions, respectively. The SMSI has been widely exploited to improve catalyst stability (Lee et al., 2015, Tang et al., 2016a, Tang et al., 2016b), identify reaction mechanisms (Matsubu et al., 2017, Bonanni et al., 2012), and enhance activity (Vannice, 1979, Sonstrom et al., 2011). It is highly desired to extend the applications of SMSI concept to broader catalyst systems, beyond metal particles, to tune catalytic properties for desirable performance.

Carbon-based catalysts have been widely used in liquid phase hydrogenation reactions because of their high chemical stability, high total surface area, and unique electronic properties (Su et al., 2017). It has been reported that the interaction between metal species and carbon surfaces can be significantly strengthened by engineering defects of, introducing functional groups to, and/or incorporating heteroatoms in carbon structures (Charlier, 2002, Lordi et al., 2001, Ebbesen and Takada, 1995). For example, single metal atoms that are anchored onto heteroatom-doped (e.g., N, O) carbon materials exhibited excellent catalytic performance for various catalytic transformations, such as methane activation (Cui et al., 2018), selective hydrogenation (Yan et al., 2015), hydrogen evolution (Fei et al., 2015, Liu et al., 2018), and oxygen reduction (Zitolo et al., 2015, Li et al., 2018). The uniformity of isolated active sites and the strong interaction between individual metal atoms and support surfaces facilitate the tuning of both selectivity and activity (Qiao et al., 2011, Liu, 2017a, Liu, 2017b, Gates et al., 2017, Wang et al., 2018, Chen et al., 2018). However, the structure of high-surface-area activated carbon supports can become extremely complex and consequently poses formidable challenges for correlating the metal-carbon interaction with the observed catalytic properties.

In this work, we fabricated hollow nanocarbons (h-NCs) that possess high-number density of surface edge/defect sites, high total surface area, and highly accessible mesopores. We anchored ∼1.0 wt.% single Pt atoms to the edge/defect sites of the h-NCs to synthesize h-NC supported Pt_1_ single-atom catalysts (SACs), denoted as 1.0 wt.% Pt_1_/h-NC. The selective hydrogenation of 3-nitrostyrene to functionalized anilines, which are industrially key intermediates for fine chemicals (Downing et al., 1997, Corma and Serna, 2006), was chosen to probe how the SMSI between carbon and single metal atoms affects the catalytic performance of the fabricated Pt_1_/h-NC SAC for liquid phase catalytic reactions. Our comprehensive characterization results indicate that the strong interaction between Pt atoms and carbon edge/defect sites modulates the electronic structure of the anchored single Pt atoms and thus their reactivity. The 1.0 wt.% Pt_1_/h-NC demonstrates excellent activity for hydrogenation of 3-nitrostyrene to 3-vinylaniline with a turnover number (TON) > 31,000/h, more than 20 times higher than that of the best catalyst for such selective hydrogenation reactions reported in the literature (Wei et al., 2014).

## Results

### Synthesis of h-NCs, Pt_1_/h-NC SACs, and Control Catalysts

**Figure 1:**
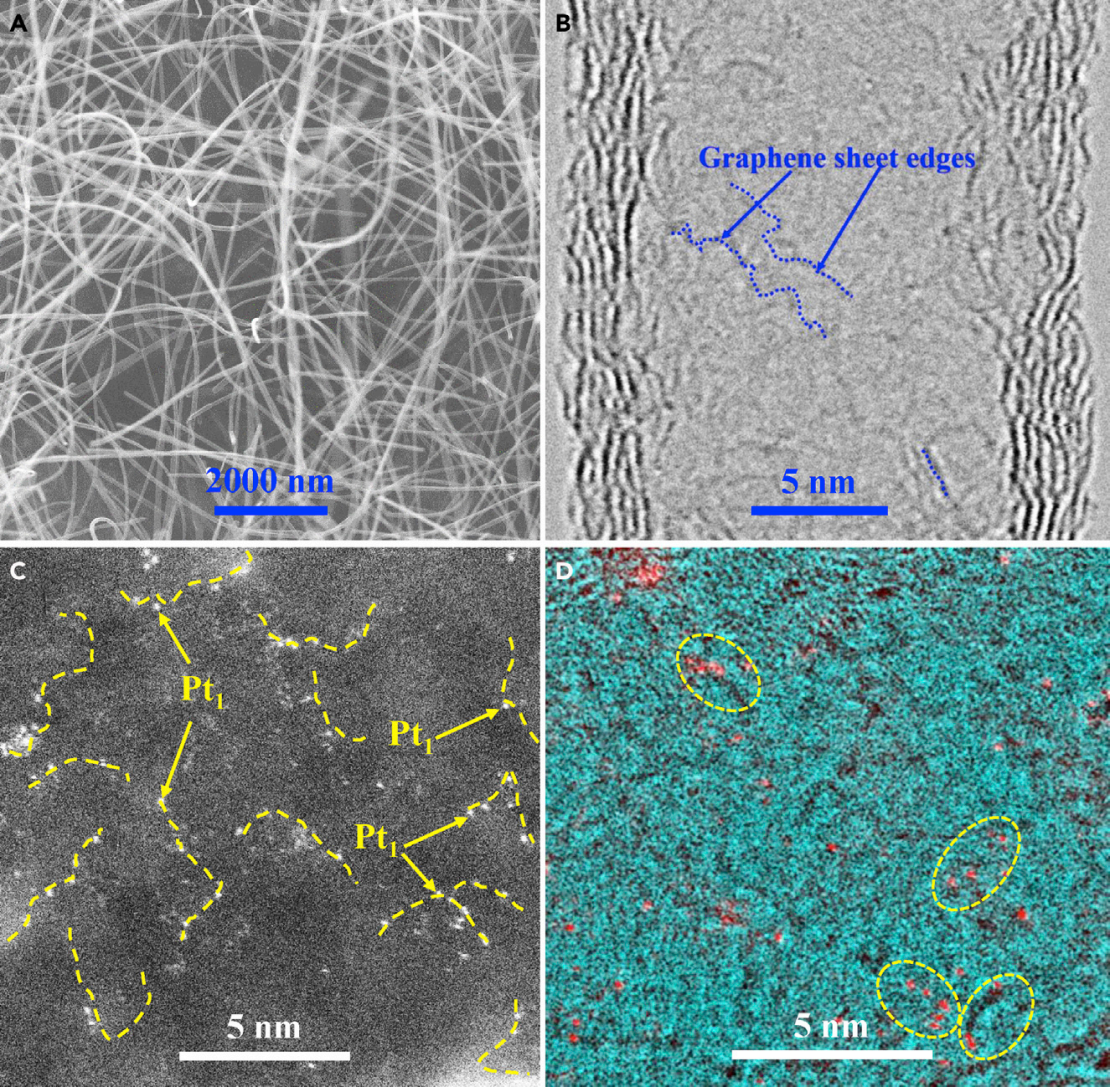
Electron Microscopy Images of Synthesized h-NCs and Fresh 1.0 wt.% Pt_1_/h-NC SAC (A) Scanning electron microscope image of morphology; (B) bright-field STEM image of edge/defect sites of the synthesized h-NCs; (C) high-magnification HAADF image of the fresh 1.0 wt.% Pt_1_/h-NC SAC; (D) false color composite image obtained from the simultaneously acquired HAADF and bright-field STEM images to show the spatial relationship between the Pt single atoms and the nanoscale graphene sheets in the h-NCs. The scale bar sizes of Figures 1A–1D are 200, 5, 5, and 5 nm, respectively.

The h-NCs were synthesized via a catalytic decomposition and reductive evaporation process by using ZnO nanowires as catalyst/template and ethanol as feedstock (see details in Transparent Methods). The residual Zn content in the synthesized h-NCs is non-detectable (<1 ppb measured by inductively coupled plasma mass spectrometry [ICP-MS]). The fabricated h-NCs possess high total surface area (1,100 m^2^/g), a tube-like morphology with inner diameters ranging from 20 to 100 nm, and lengths ranging from 1 to 20 μm (Figures 1A and S1). The wall thicknesses of the h-NCs range 2–5 nm. In addition to the large mesopores of the interior regions of the hollow tubes, the h-NCs also possess numerous small mesopores (∼3.2 nm, Figure S2) on their sidewalls. Aberration-corrected scanning transmission electron microscopy (ac-STEM) images clearly revealed numerous hyper-cross-linked graphene sheets with sizes ranging from 0.5 to 3.1 nm, representing highly disordered and defective graphene-like structures (Figures 1B and S3).

A strong electrostatic adsorption method (Qiao et al., 2015) was used to disperse Pt atoms onto the h-NCs with a nominal loading of 1.0 wt.% Pt (actual loading of 0.93 wt.% by ICP-MS). High-angle annular dark-field (HAADF) imaging technique, indispensable for characterizing metal atoms in SACs (Liu, 2017a, Liu, 2017b), was used to examine the spatial distribution of Pt atoms, clusters, and/or particles. The synthesized 1.0 wt.% Pt_1_/h-NC contained only isolated Pt single atoms without the presence of any Pt particles/clusters (Figures 1C and S4) in the h-NCs. By analyzing numerous ac-STEM images (Figure S4) obtained from different regions, we unambiguously concluded that the as-synthesized Pt_1_/h-NC catalysts contained only isolated Pt atoms uniformly dispersed onto the surfaces of the mesoporous h-NCs. The Pt atoms preferentially decorated the edges of the hyper-cross-linked nanoscale graphene sheets (Figure 1C and 1D).

A control catalyst, consisting of 1.0 wt.% Pt nanoparticles (actual loading of 0.84 wt.% by ICP-MS) with an average size of 2.3 ± 0.1 nm dispersed onto the h-NCs (Figure S5), was synthesized and denoted as 1.0 wt.% nano-Pt/h-NC. We also synthesized another control catalyst by using the carbon black Vulcan XC-72 powders. Since the BET surface area of the XC-72 (254 m^2^/g) is ∼4 times lower than that of our homemade h-NCs (1,100 m^2^/g), we loaded only ∼0.25 wt.% of Pt (actual loading of 0.08 wt.% by ICP-MS) so that the normalized number density of surface Pt atoms is approximately the same for both the Pt/h-NC and Pt/XC-72 catalysts. The STEM images (Figure S6) show that the 0.25 wt.% Pt_1_/XC-72 catalyst mainly contained atomically dispersed Pt atoms.

### Catalytic Performance and Stability of the Fabricated Pt_1_/h-NC SACs

Hydrogenation of 3-nitrostyrene is used to probe the catalytic properties of the synthesized catalysts. As shown in Table 1 and Figure S7, the 1.0 wt.% Pt_1_/h-NC SAC is highly active with a selectivity of ∼78% toward 3-vinylaniline even under very mild reaction conditions (5 bar H_2_ at 40°C). The 3-ethylaniline is the only detectable by-product. For comparison, the literature reports a selectivity of <3% (toward 3-vinylaniline) on un-modified Pt/C catalysts (Corma and Serna, 2006). The TON for 3-vinylaniline was calculated to be 31,157/h (see details on kinetic data measurements in Transparent Methods), more than 20 times higher than that of the best catalyst (0.08 wt.% Pt_1_/FeO_x_) reported in the literature (Wei et al., 2014). Under the same reaction conditions, the 1.0 wt.% nano-Pt/h-NC yielded a dramatically decreased selectivity toward 3-vinylaniline: the products consisted of ∼47% 3-vinylaniline and ∼53% 3-ethylaniline, and the TON for producing 3-vinylaniline was ∼2,018/h, about 15 times smaller than that of the 1.0 wt.% Pt_1_/h-NC SAC. Furthermore, the presence of single Pt atoms in the 1.0 wt.% nano-Pt/h-NC catalyst (Figure S5) might have contributed to the activity and selectivity of the 1.0 wt.% nano-Pt/h-NC catalyst, suggesting that the true TON and selectivity toward 3-vinylaniline on the Pt nanoparticles may be much smaller than those of the measured values. These catalytic testing results unambiguously demonstrate that the h-NC supported single Pt atoms are much more active and selective toward 3-vinylaniline. Our experimental results clearly show that the h-NC supported Pt nanoparticles produce dominantly 3-ethylaniline (∼53%), whereas the graphene-edge-anchored Pt single atoms yield dominantly 3-vinylaniline (∼78%). The large differences in product selectivity manifest the intrinsically different catalytic properties of strongly anchored Pt single atoms from those of the supported Pt nanoparticles.

**Table 1:** Chemoselective Hydrogenation of 3-Nitrostyrene on Pt/h-NC Catalysts Compared with Most Active Catalysts Reported in the Literature

Sample	T/°C	Time/min	Conv (%)	Sel (%)	TON^a^	Reference
1.0 wt.% Pt_1_/h-NC SAC	40	10	97.1	77.8	31,157	This work*
1.0 wt.% nano-Pt/h-NC	40	10	93.8	47.1	2,018	This work*
Pure h-NCs	40	60	0	0	0	This work*
0.25 wt.% Pt/XC-72	40	10	9.7	41.3	1,225	This work*
Pure XC-72	40	60	0	0	0	This work*
0.08 wt.% Pt_1_/FeO_x_	40	50	96.5	98.6	1,493	Wei et al., 2014
0.2 wt.% Pt/TiO_2_	40	390		93.1	56	Serna et al., 2009
0.01 wt.% Pt/TiO_2_	85	360		48.4	1,449	Serna et al., 2009
1.4 wt.% Pt/ZnO	75	/		97	1,560	Berguerand et al., 2015
Commercial Pt/C^b^	75	/	/	0	0	Berguerand et al., 2015

*Reaction conditions: H_2_ pressure = 5 bar; Pt/substrate = 0.078%; 8 mL reaction mixture: 0.5 mmol 3-nitrostyrene, ethanol as solvent, O-xylene as internal standard.

a The amount of the produced 3-vinylaniline molecules per active site per hour. The turnover number (TON) value was measured by keeping the substrate conversion below 25% by tuning the molecular ratio of Pt atoms to 3-nitrostyrene molecules.

b The Pt loading of the commercial Pt/C catalyst is 1.0 wt.% (Berguerand et al., 2015).

For the atomically dispersed 0.25 wt.% Pt_1_/XC-72 control catalyst, the selectivity toward 3-vinylaniline was measured to be ∼41% and the corresponding TON was 1,225/h, ∼25 times lower than that on the 1.0 wt.% Pt_1_/h-NC SAC. The significant changes in both the TON and selectivity implies that the catalytic property of the 1.0 wt.% Pt_1_/h-NC SAC is intrinsically different from that of the atomically dispersed 0.25 wt.% Pt_1_/XC-72. As clearly shown in Table 1, the TON for 3-vinylaniline on the 1.0 wt.% Pt_1_/h-NC SAC is at least one order of magnitude higher than that on all other catalysts. Control experiments conducted on pure h-NC and XC-72 supports only, under the same reaction conditions, did not exhibit any detectable products.

For the used 1.0 wt.% Pt_1_/h-NC SAC, most of the single Pt atoms (∼99%) were still isolated and only very few small aggregates of loosely connected Pt atoms (∼0.4 nm) were detected (Figure S8). These atomically dispersed aggregates do not seem to form Pt-Pt bond since the distances among these Pt atoms are much larger than that of crystalline Pt clusters (Yang et al., 2015). The fact that the Pt single atoms remained isolated after the catalytic reaction suggests that the isolated single Pt atoms were strongly anchored onto the edge/defect sites of the h-NCs. Moreover, atomic resolution HAADF-STEM images show that the individual Pt atoms primarily decorated the edges/steps of nanoscale pieces of graphene sheets (indicated by the green dash lines in Figures 1D and S4) present in the h-NCs. These edge carbon atoms strongly interact with the individual single Pt atoms as suggested by density functional theory (DFT) calculations (Wei et al., 2017a). Since the XC-72 carbon support is highly graphitic and does not possess many strong anchoring edge/defect sites, the originally atomically dispersed Pt atoms in the XC-72 carbon sintered into Pt nanoparticles/clusters with an average size of 1.1 ± 0.1 nm after one cycle of selective hydrogenation of 3-nitrostyrene (Figure S9). These results suggest that, although the Pt was atomically dispersed in the fresh Pt/XC-72 catalyst, these Pt atoms did not strongly interact with the XC-72 carbon and thus sintered during the hydrogenation reaction. The XC-72 carbon and the h-NCs are structurally very different, resulting in different degrees of Pt-carbon interactions and consequently the differences in catalytic performance.

### Characterization of the Edge Site Defects in the Fabricated Pt1/h-NC SACs

To understand why the synthesized h-NCs strongly anchored Pt single atoms, Raman spectroscopy, capable of providing the density of defects of carbon materials (Ferrari and Robertson, 2000, Sadezky et al., 2005), was used to evaluate the properties of the carbon supports. By using the empirical formula models (Cançado et al., 2006, Tuinstra and Koenig, 1970), the defect density of h-NCs was estimated to be about five times higher than that of the XC-72 carbon (Figure S10 and Table S1), which suggests that the synthesized h-NCs consist of numerous defects (edge sites) as evidenced in STEM images. After Pt single atoms were deposited on the h-NCs, the defect density decreased by ∼17% (Table S1). Based on the STEM images (Figures 1D and S8) as well as the spectroscopy data we propose that the drop of the estimated defect density by Raman spectroscopy is caused by the strong anchoring of Pt atoms to the edge/defect sites of the nanoscale graphene sheets. DFT calculations suggested that single Pt atoms can directly bond with graphene edges with a binding energy as high as 7.46 eV (Wei et al., 2017a). After Pt single atoms were deposited on the XC-72 carbon, the defect density slightly decreased by ∼6%, suggesting that the XC-72 carbon cannot strongly anchor the atomically dispersed Pt atoms, which corroborates the STEM results that the atomically dispersed Pt atoms sintered to particles (∼1.1 nm) after the hydrogenation reaction.

### The Electronic Structure of the Fabricated Pt_1_/h-NC SACs

**Figure 2:**
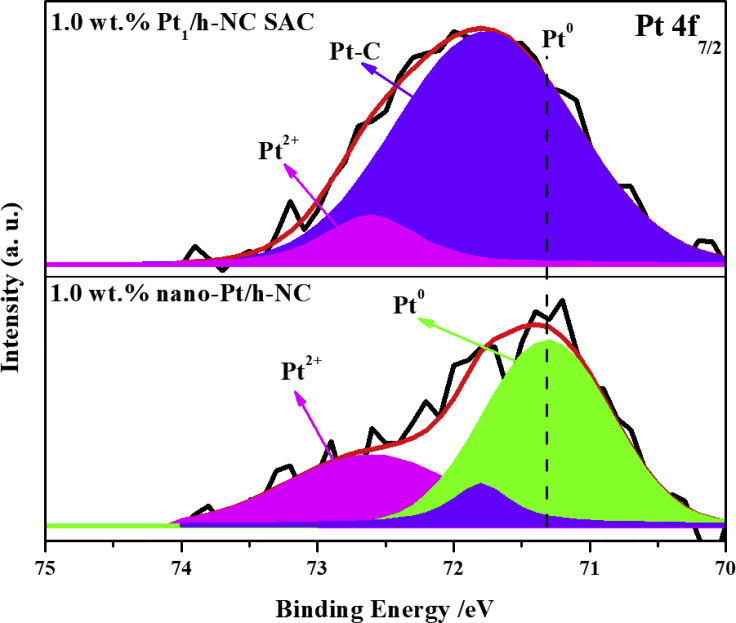
Electronic Structures of h-NCs and h-NC Supported Pt Catalysts Pt 4f 7/2 XPS of 1.0 wt.% Pt_1_/h-NC SAC and 1.0 wt.% nano-Pt/h-NC.

The X-ray photoelectron spectroscopy (XPS) C1s spectrum of the 1.0 wt.% Pt_1_/h-NC SAC shows that, in addition to the carbon sp^2^ (284.7 eV) and sp^3^ (285.2 eV) peaks, there is a new component located at 284.3 eV (Figure S11A), assignable to a Pt-C bond originating from a strong covalent interaction between Pt atoms and the under-coordinated carbon atoms of the h-NCs (Rajasekaran et al., 2012, Ng et al., 2010). XPS spectra (Figure 2) suggest that the 1.0 wt.% Pt_1_/h-NC SAC contains only ∼10% of Pt^2+^ species, whereas ∼90% of the Pt species possess a binding energy of 71.8 eV, higher than that of the metallic Pt species (71.3 eV) but lower than that of the Pt^2+^ species (72.6 eV). We denote these Pt species as Pt^δ+^ (0<δ < 2). The occurrence of the Pt^δ+^ (71.8 eV) species can be attributed to the formation of Pt-C bonds with electron transfer from the Pt single atoms to the carbon support (Alderucci et al., 1995, Axnanda et al., 2015). The presence of the Pt^2+^ species may result from the oxidation by the oxygen-containing groups present on the surfaces of the h-NCs as evidenced by the O 1s spectrum (Figure S11B), which shows three components assignable to the Pt-OH (532.1 eV, 60%), Pt-H_2_O (533.1 eV, 37%), and Pt-O species (530.0 eV, 3%) (Alderucci et al., 1995). The STEM images, Raman spectra, and XPS data all support the conclusion that the Pt single atoms decorated the edges of the cross-linked nanoscale graphene sheets in the h-NCs (Figures 1D and S4) and directly bonded with the edge carbon atoms to form strong Pt-C bonds (Rajasekaran et al., 2012, Ng et al., 2010), probably arising from the hybridization of Pt 5d and the abundant C 2p orbitals of the edge carbon atoms (Tang et al., 2011).

The Pt 4f XPS spectrum of the 1.0 wt.% nano-Pt/h-NC (Figure 2) exhibits mostly metallic Pt species (71.3 eV, 61%), an appreciable amount of Pt^2+^ (72.6 eV, 30%) and a minor amount of Pt^δ+^ (71.8 eV, 9%). The XPS C1s spectrum of the 1.0 wt.% nano-Pt/h-NC (Figure S11A) shows that, in addition to the carbon sp^2^ (284.7 eV) and sp^3^ (285.2 eV) peaks, there is also a very weak peak at 284.3 eV, suggesting the formation of a minor amount of Pt-C bond, which may originate from the interaction of the edge atoms of the Pt nanoparticles with the carbon support (Rajasekaran et al., 2012, Ng et al., 2010). The Pt 4f XPS spectrum of the 0.25 wt.% Pt_1_/XC-72 catalyst could not be quantitatively analyzed because of the signal-to-noise problem (Yang et al., 2015, Zhang et al., 2016). But the oxidation state of the atomically dispersed Pt atoms in the 0.25 wt.% Pt_1_/XC-72 catalyst seems to be between +2 and +4 (Figure S12), suggesting that the Pt species may primarily be in the form of highly dispersed Pt oxides. The absence of an obvious Pt-C peak (Figure S13) in the 0.25 wt.% Pt_1_/XC-72 suggests that the atomically dispersed Pt atoms did not interact strongly with the graphitic carbon support, resulting in sinter of Pt atoms and small clusters during the hydrogenation reaction, corroborating the conclusion from the STEM imaging observation (Figure S9) and Raman spectroscopy data (Figure S10).

### Effects of Strong Pt-Carbon Interaction on Catalytic Performance

The geometric and electronic properties of active metal species play a crucial role in determining the adsorption behavior of functional groups and the dissociation behavior of H_2_ (Armbrüster et al., 2012, Blaser et al., 2009, Wei et al., 2014, Wei et al., 2017b, Lou et al., 2018). Pt metals show excellent catalytic capability of H_2_ dissociation with a reaction barrier as low as 0 and 0.067 eV on Pt (001) and Pt (111) surfaces, respectively (Pasteur et al., 1997, Olsen et al., 1999). The strength of the Pt-H bonds is too high (even close to H-H in H_2_) to be efficient and chemoselective for catalytic reactions (Ludwig et al., 2006, Corma et al., 2008). For selective hydrogenation to targeted products, a catalyst that possesses facile activation of H_2_ and weak adsorption strength of the dissociated H atoms can exhibit optimal efficiency in balancing activity and selectivity for the targeted product (Lucci et al., 2015a, Lucci et al., 2015b, Lucci et al., 2016, Kyriakou et al., 2012). For the Pt_1_/h-NC SAC system, the metal-carbon interaction results in electron transfer from the Pt atoms to the carbon support and they most probably maintain their valence state under the hydrogenation reaction conditions. The electron depletion of the edge-carbon-anchored Pt atoms induces downshift of the d-band center of the Pt^δ+^ species (0< δ < 2) and consequently reduces, when compared with the metallic Pt species, the binding strength of the dissociatively adsorbed hydrogen atoms (Armbrüster et al., 2012, Namba et al., 2018). Such a change of the d-band center of the single Pt species facilitates the activation of H_2_ and thus provides more activated and weakly bound hydrogen species to accelerate the catalytic reaction. Under hydrogenation reaction conditions, the nanometer-sized Pt oxide species can be easily reduced to the metallic state and sintered to Pt particles (Huizinga et al., 1984, Duan et al., 2018), which will strongly bind the adsorbed H species, resulting in the lack of weakly bound hydrogen species for catalytic reactions. Furthermore, the H_2_ dissociation over Pt_1_/h-NCs may follow the heterolytic dissociation procedure to yield H^δ+^ and H^δ−^ species on the Pt-carbon interface, which may significantly enhance the catalytic activity of hydrogenation reactions as reported in the literature (Liu et al., 2016, Wang et al., 2016, Dhiman and Polshettiwar, 2018). The spillover effects of the activated H species over the carbon support cannot be ruled out. The influence of such spillover H species on the observed activity and selectivity on hydrogenation reactions is not clear and needs to be further investigated. Based on our characterization and catalytic testing data, we propose that the strong Pt-carbon interaction in the Pt_1_/h-NCs significantly modifies the electronic structure of the carbon-edge-anchored single Pt atoms and correspondingly their catalytic properties for H_2_ activation. The enhanced electronic SMSI makes the 1.0 wt.% Pt_1_/h-NC SAC a much more highly active catalyst than that of the atomically dispersed 0.25 wt.% Pt_1_/XC-72 and 1.0 wt.% nano-Pt/h-NC catalysts for 3-nitrostyrene hydrogenation.

## Discussion

The selective hydrogenation of the C=C bonds is considered to be highly sensitive to the size of the Pt ensembles (Wei et al., 2014, Corma et al., 2008, Lucci et al., 2015a, Lucci et al., 2015b). On the other hand, the selectivity for nitro groups is independent of the size of the Pt particles (Corma et al., 2008, Peng et al., 2018). It has been reported that Pt single atoms especially prefer the adsorption of nitro groups to the C=C groups (Wei et al., 2014, Peng et al., 2018). The dissociated H species may spillover via carbon support to the nitro groups that are activated by single Pt atoms to complete the catalytic reaction cycle (Wei et al., 2014). Hence, the strong Pt_1_-C interaction in the 1.0 wt.% Pt_1_/h-NC SAC not only enhances H_2_ activation to provide more activated and weakly bound hydrogen species but also provides stable single sites that inhibit the C=C activation and simultaneously enhances the nitro group activation. The strong electronic Pt-carbon interaction not only enhances anchoring of single Pt atoms to the edge carbon atoms of the h-NCs but also significantly modifies their catalytic properties for selective hydrogenation. As a result of the strong Pt_1_-carbon interaction the Pt atoms transfer electrons to carbon support to form Pt^δ+^ species, which are proposed to be the active sites for facilely dissociating H_2_ and preferential adsorption of the specific sites of the reactant molecules. The fabricated Pt_1_/h-NC SACs exhibited excellent catalytic performance for selective hydrogenation of 3-nitrostyrene to 3-vinylaniline because of the covalent bonding of Pt_1_ to the edge carbon atoms of the nanoscale graphene pieces in the synthesized h-NCs. Although further improvement in designing the carbon structure to tune the Pt-carbon interaction may result in further improved selectivity, our research work clearly demonstrated the power in utilizing the electronic SMSI in SACs to significantly enhance the activity for desired reaction products. The strategy utilized in this work is general and can be broadened to construct different catalytic systems, especially SACs, for a plethora of catalytic reactions, including liquid- and gas-phase transformation of important molecules.

### Limitations of Study

Our research on electronic strong metal-carbon interaction in SACs has extended the SMSI concept to broader catalyst systems for tuning catalytic properties of strongly anchored single metal atoms. A combined density functional theory and Monte Carlo approach for quantifying the metal-carbon interactions at the reaction temperatures can provide an in-depth understanding on the electronic strong metal-carbon interaction in SACs and may provide guidance for designing better catalysts for liquid-phase transformation of important molecules. The reaction mechanisms of selective 3-nitrostyrene hydrogenation, the spillover effects of the activated H species, and the role of the highly defective carbon structures in the adsorption of reactant molecules need to be further investigated. Although the study of these important questions on the fundamental understanding of selective hydrogenation of important complex molecules is beyond the scope of this current work, such fundamental studies can provide guidance on effectively utilizing the strong metal-carbon interaction to design robust SACs with desired catalytic performance.

## Methods

All methods can be found in the accompanying Transparent Methods supplemental file.

## Supplemental Information

Document S1. Transparent Methods, Figures S1–S13, and Table S1
