# Supplementary material for: Nanocarbon-Edge-Anchored High-Density Pt Atoms for 3-nitrostyrene Hydrogenation: Strong Metal-Carbon Interaction
Source: iScience. 2019 Feb 20;13:190–8. doi: 10.1016/j.isci.2019.02.016 (PMC6409412; doi:10.1016/j.isci.2019.02.016)
Supplement: Document S1. Transparent Methods, Figures S1–S13, and Table S1 [file mmc1.pdf]

ISCI, Volume *13*

Supplemental Information

Nanocarbon-Edge-Anchored High-Density Pt Atoms for 3-nitrostyrene Hydrogenation: Strong Metal-Carbon Interaction

Yang Lou, Honglu Wu, and Jingyue Liu

SUPPLEMENTAL INFORMATION

Nanocarbon-Edge-Anchored High-Density Single Pt Atoms for Selective Hydrogenation of 3-nitrostyrene: Strong Metal-Carbon Interaction

Yang Lou[1], Honglu Wu[1], Jingyue Liu[1, 2]*

[1]Department of Physics, Arizona State University, Tempe, Arizona 85287, United States

[2]Lead contact

*Correspondence: jingyue.liu@asu.edu

Contents:

Figure S1 The SEM images (a-c) and Aberration-corrected HADDF-STEM image (d) of h-NCs.

Figure S2 The pore size distribution of h-NCs.

Figure S3 The Aberration-corrected STEM images of h-NCs: Aberration-corrected STEM images of h-NCs: the size distribution of graphene sheets (a-c).

Figure S4 Low-magnification and high-magnification aberration-corrected HAADF/STEM images of fresh 1.0 wt.% Pt_1/ h-NC SAC (a-f); schematic diagram for single Pt atoms anchored on the edge defects of graphene sheets of hollow nanocarbon (g).

Figure S5 Low-magnification and high-magnification aberration-corrected HAADF/STEM images of 1.0 wt.% nano-Pt/ h-NC.

Figure S6 Low-magnification and high-magnification aberration-corrected HAADF/STEM images of atomically dispersed fresh 0.25 wt.% Pt_1/XC-72.

Figure S7 Time-dependent conversion and selectivity of 1.0 wt.% Pt_1/h-NC SAC, 1.0 wt.% nano-Pt/h-NC and 0.25 wt.% Pt_1/XC-72 for 3-nitrostyrene selective hydrogenation.

Figure S8 Low-magnification and high-magnification aberration-corrected HAADF/STEM images of the used 1.0 wt.% Pt_1/ h-NC SAC.

Figure S9 The Pt particle size distribution and HAADF/STEM images of used 0.25 wt.% Pt_1/XC-72.

Figure S10 Raman spectra of carbon and carbon supported Pt catalysts.

Figure S11 XPS of h-NCs and h-NC supported Pt catalysts: C 1s spectra (a) and O 1s spectra (b).

Figure S12 XPS Pt 4f spectra of 0.25 wt.% Pt_1/XC-72.

Figure S13 XPS C 1s spectra of: 1.0 wt.% Pt_1/h-NC SAC, 0.25 wt.% Pt_1/XC-72 and pure XC-72.

Table S1 Summary of the defect density and crystallite size of the synthesized Pt SACs and nano-Pt catalysts.

Transparent Methods

Supplemental References

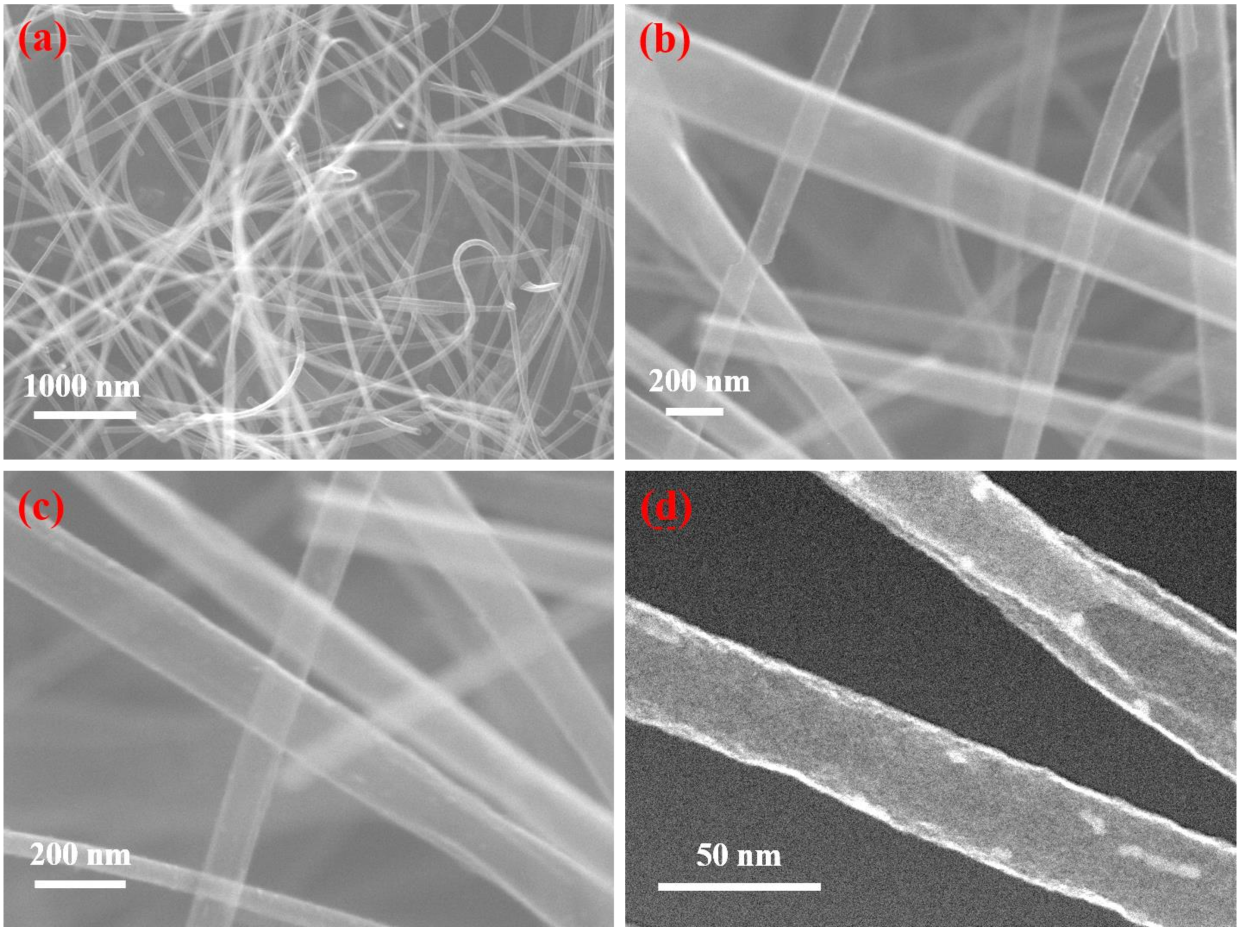

Figure S1 SEM images (a-c) and aberration-corrected HAADF-STEM image (d) of h-NCs show the general morphology and the wall thicknesses of the synthesized hollow nanocarbon tubes, Related to Figure 1.

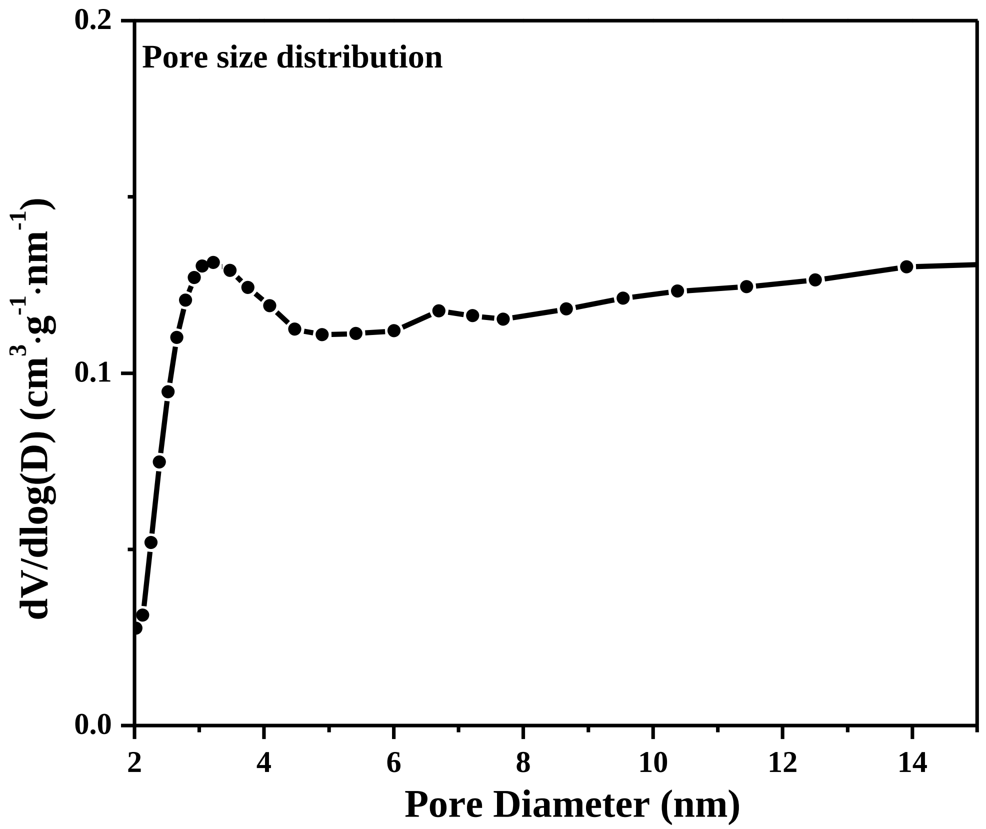

Figure S2 Pore size distribution of the synthesized h-NCs, Related to Figure 1.

The average size of the pores on the wall of the h-NCs was estimated to be 3.2 nm.

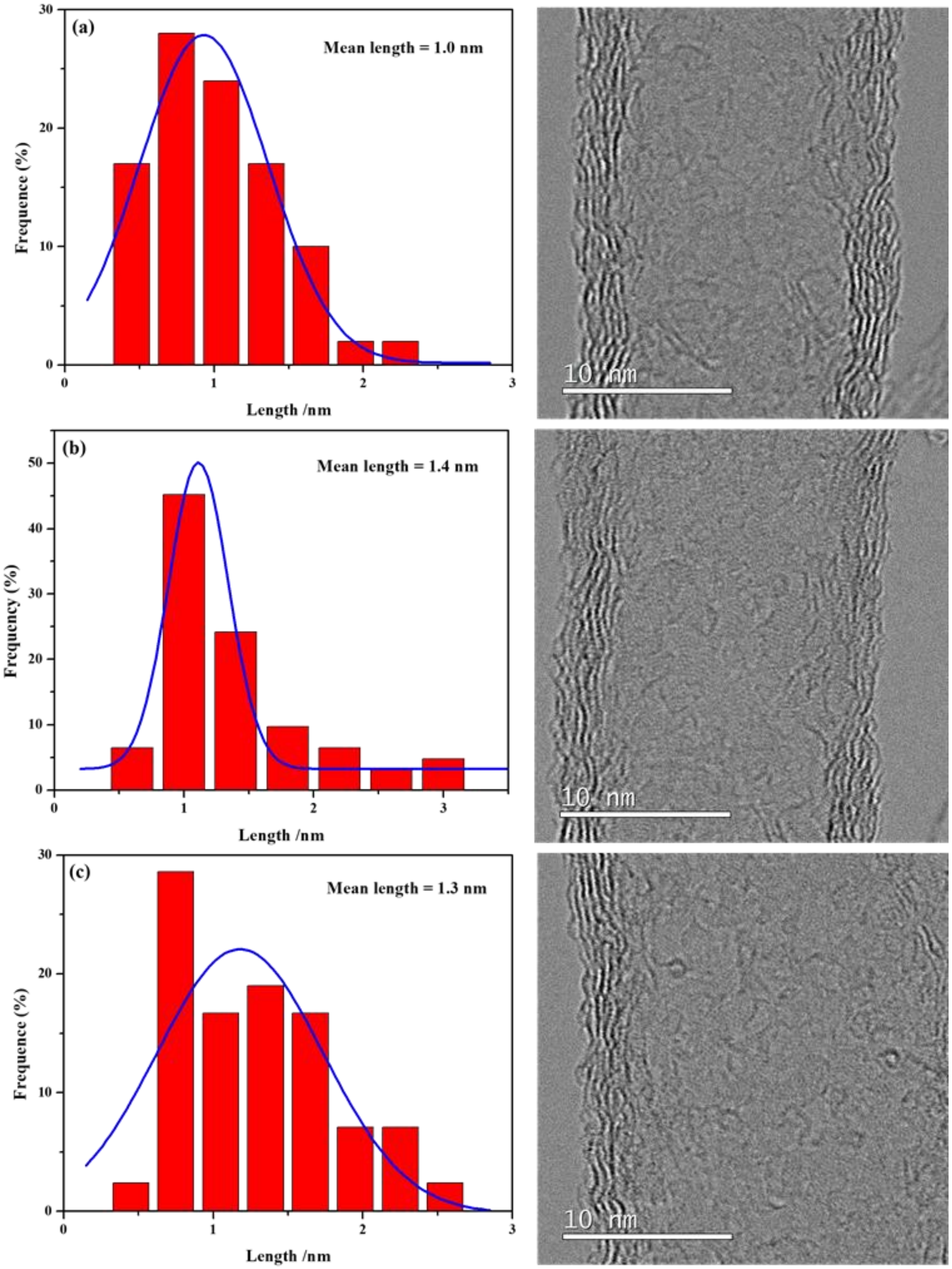

Figure S3 Aberration-corrected STEM images of h-NCs and the size distributions of the corresponding nanoscale graphene sheets (a-c), Related to Figure 1.

The synthesized h-NCs are composed of stacking of hyper-cross-linked graphene sheets, resulting in highly disordered structure, high surface area and tube-type morphology. The hyper-cross-linked graphene sheets exhibit high number density edge sites (Figure S3a-S3c). The lateral sizes of the graphene sheets range from 0.5 nm to 3.1 nm with an average length of 1.2 ± 0.2 nm. The histograms were obtained by measuring the identifiable lengths of the graphene sheets in the corresponding bright-field STEM images.

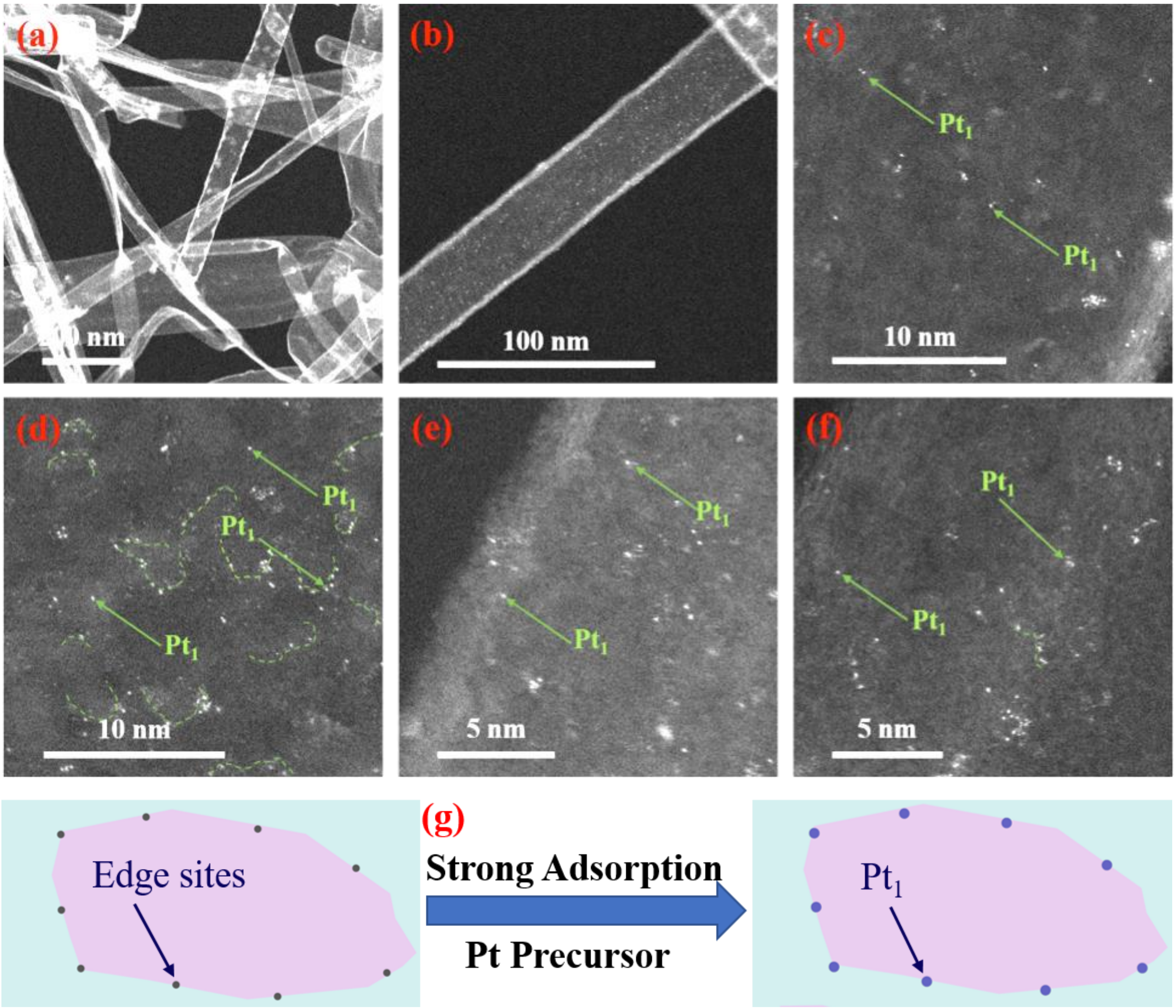

Figure S4 Low-magnification and high-magnification aberration-corrected HAADF/STEM images of fresh 1.0 wt.% Pt_1/h-NC SAC (a-f); schematic diagram for single Pt atoms anchored on the edges of graphene sheets of h-NCs (g), Related to Figure 1.

As clearly shown in Figure S4 (a), low-magnification HAADF-STEM image confirms the absence of any Pt particles or clusters in the fresh 1.0 wt.% Pt_1/h-NC SAC. The high-magnification HAADF-STEM images (Figure S4 (b-f)) confirm that the Pt atoms are atomically dispersed on the surfaces of the h-HC support. By examining numerous low/high magnification HAADF-STEM images (Figure S4 (a-f)) obtained from different regions of the synthesized h-NCs, we unambiguously concluded that the fresh1.0 wt.% Pt_1/h-NC SAC contains only isolated Pt atoms without the presence of any Pt particles or clusters. Moreover, Pt atoms are mainly decorated on the edges of those hyper-cross-linked graphene sheets. A small amount of single Pt atoms may have trapped between the layers of the graphene sheets (Yan et al., 2015; Tang et al., 2016). However, since the sizes of the graphene sheets are so small and there are almost no observable close stacking of parallel graphene sheets in the synthesized h-NCs the Pt atoms should not be stably trapped between layers of graphene. Figure S4 (g) is the schematic diagram to illustrate the anchoring of single Pt atoms onto the edges of nanoscale graphene sheets in the synthesized h-NCs.

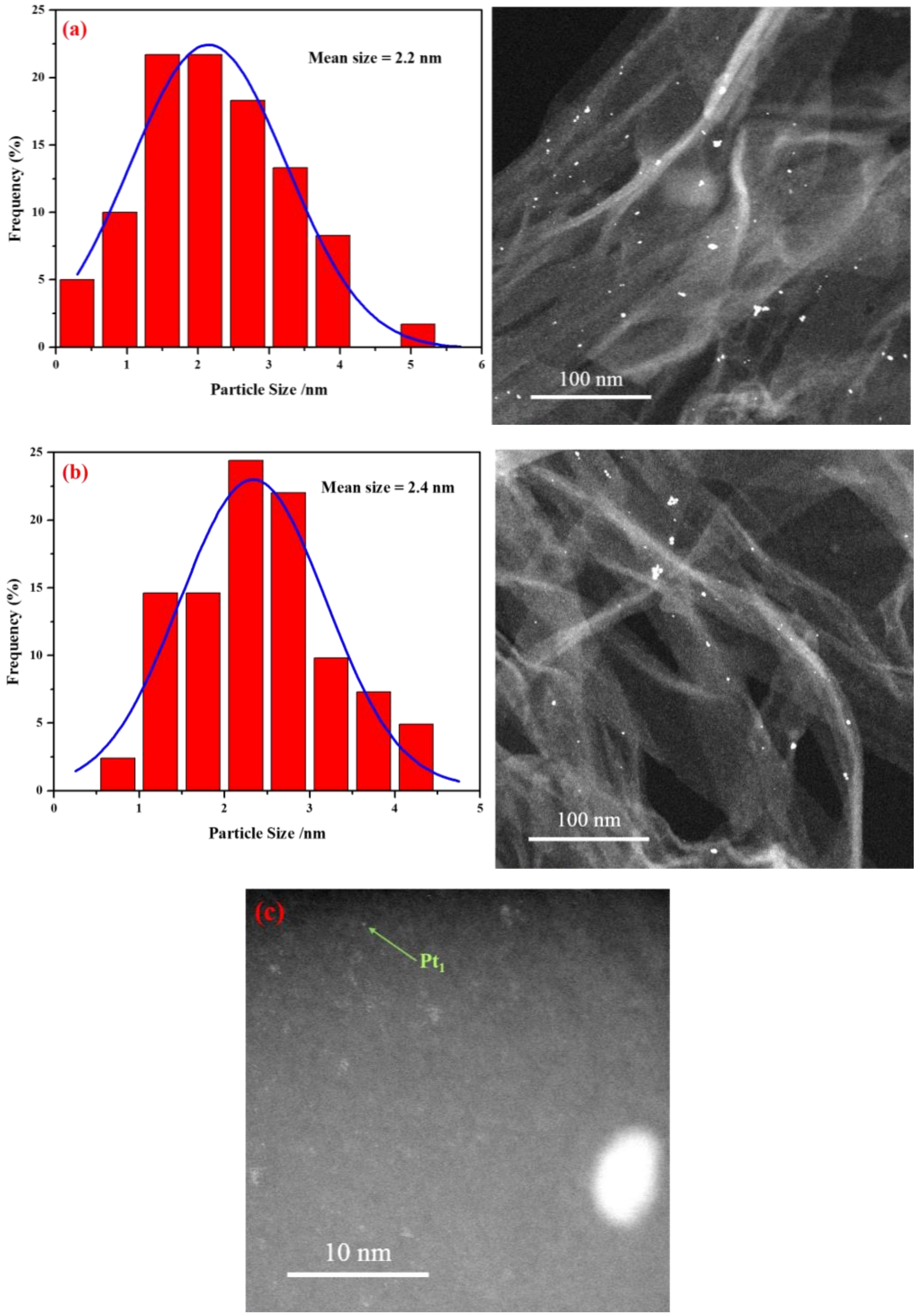

Figure S5 Low-magnification and high-magnification aberration-corrected HAADF/STEM images of 1.0 wt.% nano-Pt/h-NC, Related to Figure 1. The average particle size is 2.3 ± 0.1 nm.

Pt particles of 1.0 wt.% nano-Pt/h-NCs are distributed on the surfaces of the h-NCs (Figure S5 a-b) and the average size of the Pt particles was measured to be 2.3 ± 0.1 nm. From the high-magnification image (c), we can see the presence of some single Pt atoms in the 1.0 wt.% nano-Pt/h-NC catalyst. The presence of these anchored Pt single atoms may make appreciable contribution to the experimentally measured activity and selectivity for 3-nitrostyrene hydrogenation over the 1.0 wt.% nano-Pt/h-NC catalyst.

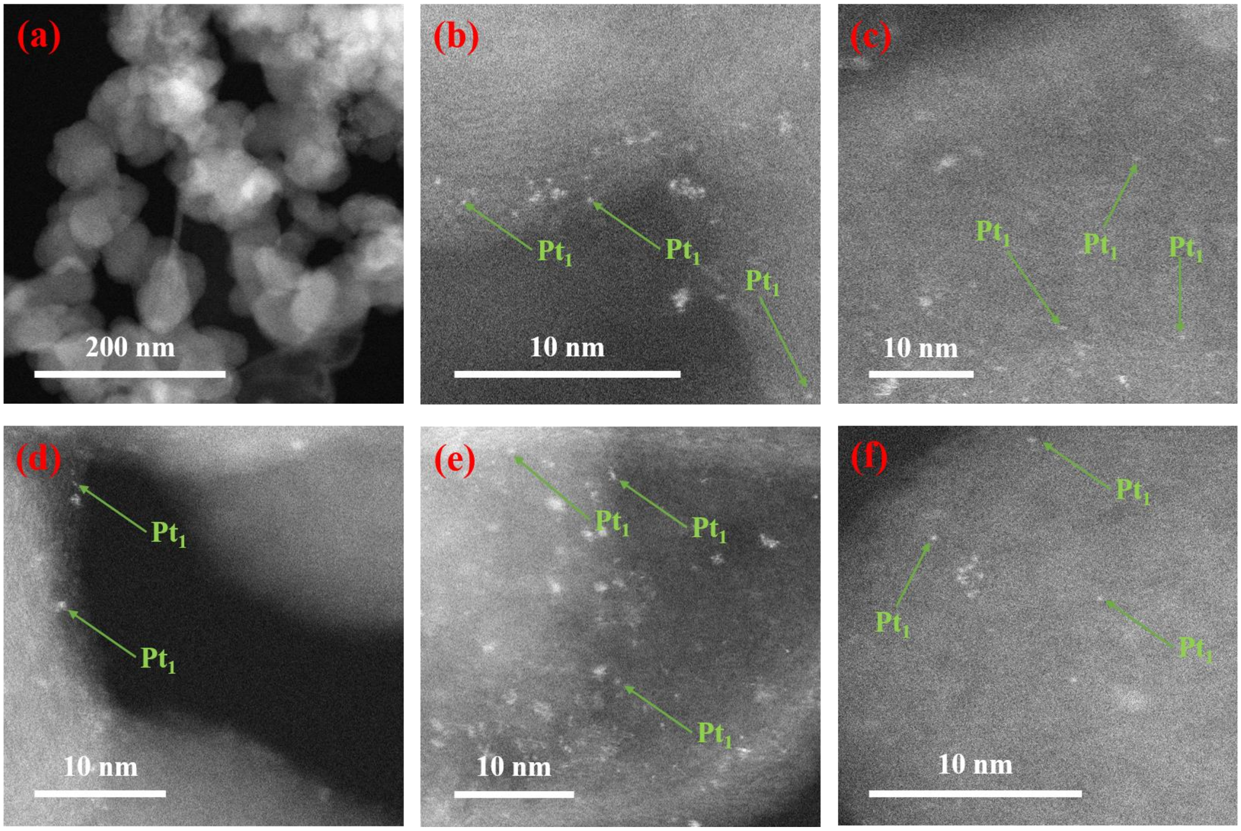

Figure S6 Low-magnification and high-magnification aberration-corrected HAADF/STEM images of an as-synthesized atomically dispersed 0.25 wt.% Pt_1/XC-72 catalyst, Related to Figure 1.

Figure S6 (a) and numerous other low-magnification HAADF-STEM images confirm the absence of any Pt particles or clusters in the fresh 0.25 wt.% Pt_1/XC-72 catalyst. The high-magnification HAADF-STEM images (Figure S6 (b-f)) confirm that the Pt atoms are atomically dispersed on the surfaces of the XC-72 support. By examining numerous low/high magnification HAADF-STEM images (Figure S6 (a-f)) obtained from different regions, we unambiguously concluded that the as-prepared 0.25 wt.% Pt_1/XC-72 contains only isolated Pt atoms and atomically dispersed small Pt clusters of a few Pt atoms without the presence of Pt particles.

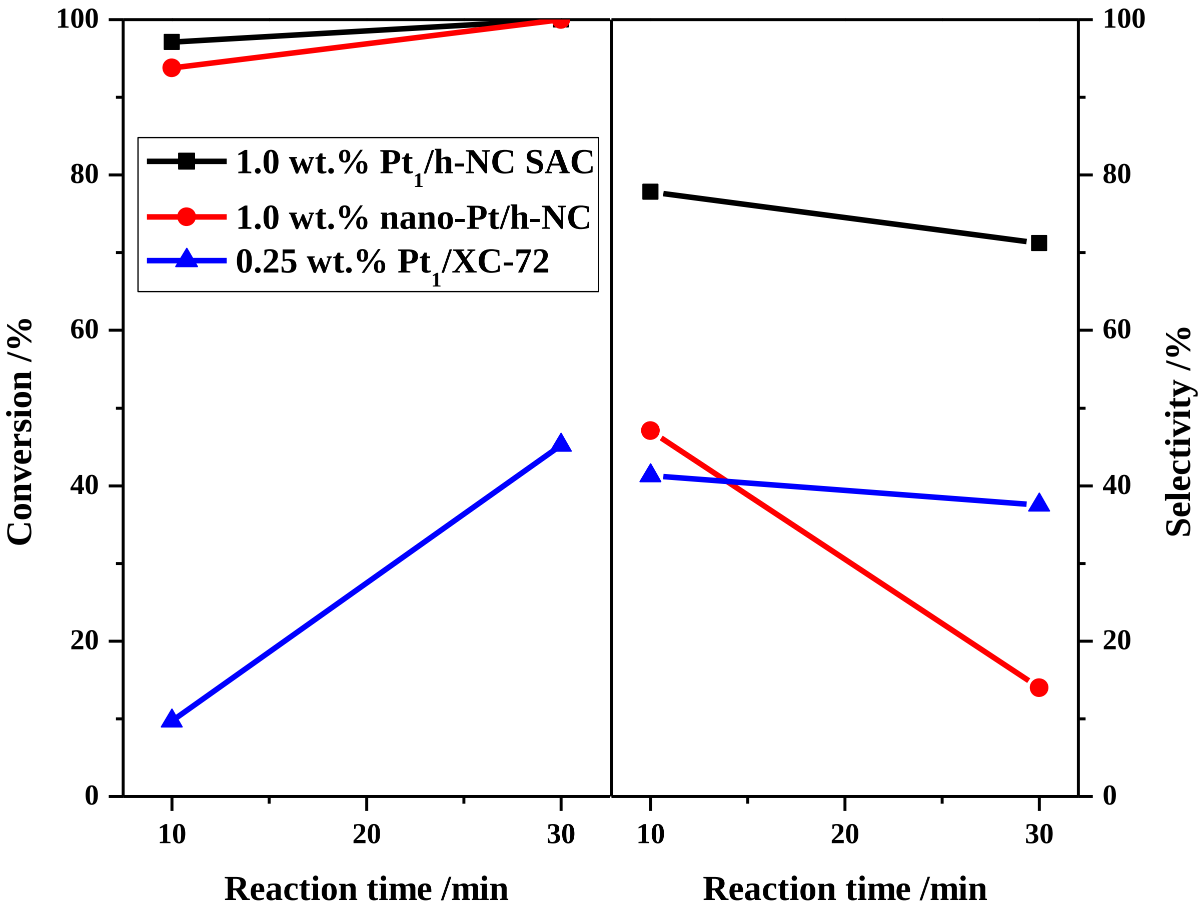

Figure S7 Time-dependent conversion and selectivity of 1.0 wt.% Pt_1/h-NC SAC, 1.0 wt.% nano-Pt/h-NC and 0.25 wt.% Pt_1/XC-72 for selective hydrogenation of 3-nitrostyrene, Related to Table 1.

With the increase of reaction time, the catalytic conversion of 3-nitrostyrene over 1.0 wt.% Pt_1/h-NC SAC, 1.0 wt.% nano-Pt/h-NC and 0.25 wt.% Pt_1/XC-72 gradually increases but the selectivity toward the 3-vinylaniline gradually decreases. Further hydrogenation of the produced 3-vinylaniline to 3-ethylaniline is possible but the formation of small Pt clusters or particles with reaction time also decreases the selectivity toward 3-vinylaniline. Promoters to either boost the activity or selectivity may need to be developed to increase the yield of the final product.

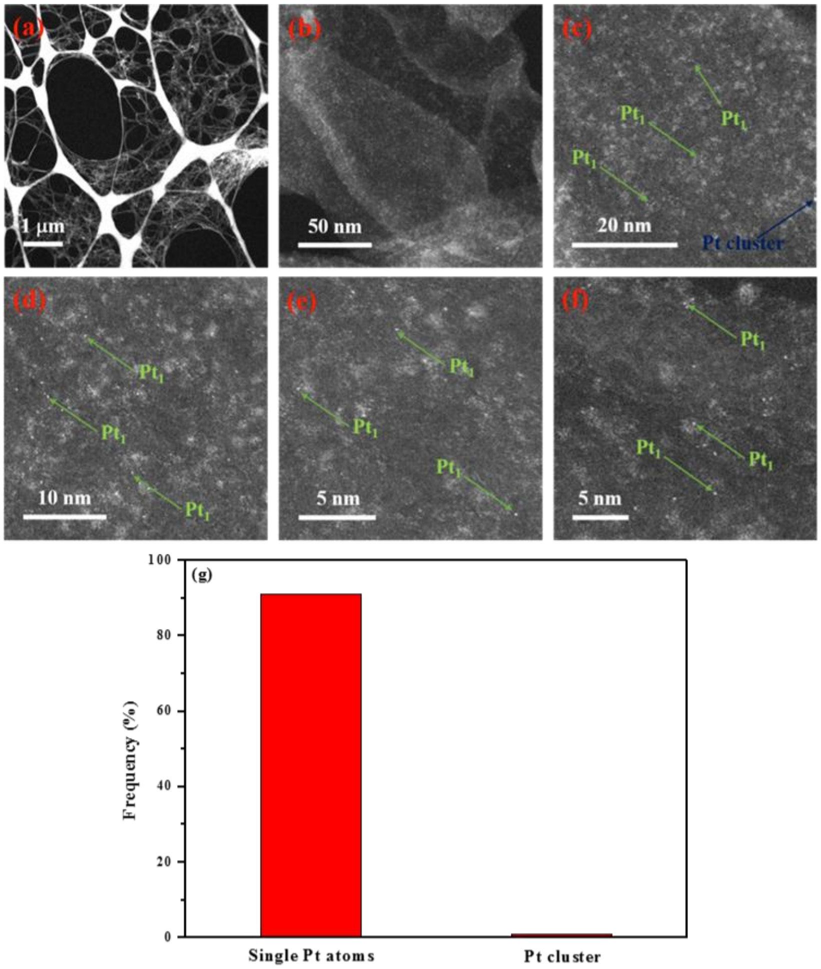

Figure S8 Low-magnification and high-magnification aberration-corrected HAADF/STEM images of used 1.0 wt.% Pt_1/h-NC SAC, Related to Figure 1. The 1.0 wt.% Pt_1/h-NC SAC was used for 30 min at a reaction temperature of 40 ^oC.

As clearly shown in Figure S8 (a-b), low-magnification HAADF-STEM images did not show Pt particles in the used 1.0 wt.% Pt_1/h-NC SAC. Only the presence of very few Pt clusters (around 0.4-0.5 nm) are observable (Figure S8 b). The high-magnification HAADF-STEM images (Figure S8 (c-f)) confirm that the used catalyst consists primarily of isolated single Pt atoms uniformly dispersed on the surfaces of the h-NCs.

In order to quantify the amount of the small Pt clusters present in the used 1.0 wt.% Pt_1/h-NC SAC, we conducted a statistical analysis of the number of Pt single atoms and small clusters (Figure S8 c) and the result is shown in Figure S8 g. We estimated that ~99% of the Pt in the used 1.0 wt.% Pt_1/h-NC SAC remained as isolated single Pt atoms after the hydrogenation reaction. Only ~1% of the Pt single atoms in the fresh catalyst sintered to atomically dispersed, loosely connected Pt clusters without the formation of Pt-Pt bonding (Yang et al., 2015). These results suggest that all the Pt atoms were still atomically dispersed after the catalytic hydrogenation reaction. Such stable SACs assure that the measured catalytic activity and kinetic data originated from the anchored single Pt atoms rather than from Pt clusters/particles.

By examining numerous low/high magnification HAADF-STEM images (Figure S8 (a-f)) obtained from different regions, we unambiguously concluded that the used 1.0 wt.% Pt_1/h-NC SAC still predominately consists of isolated Pt atoms (99%). However, after three cycles, many of the Pt atoms aggregated to form Pt clusters.

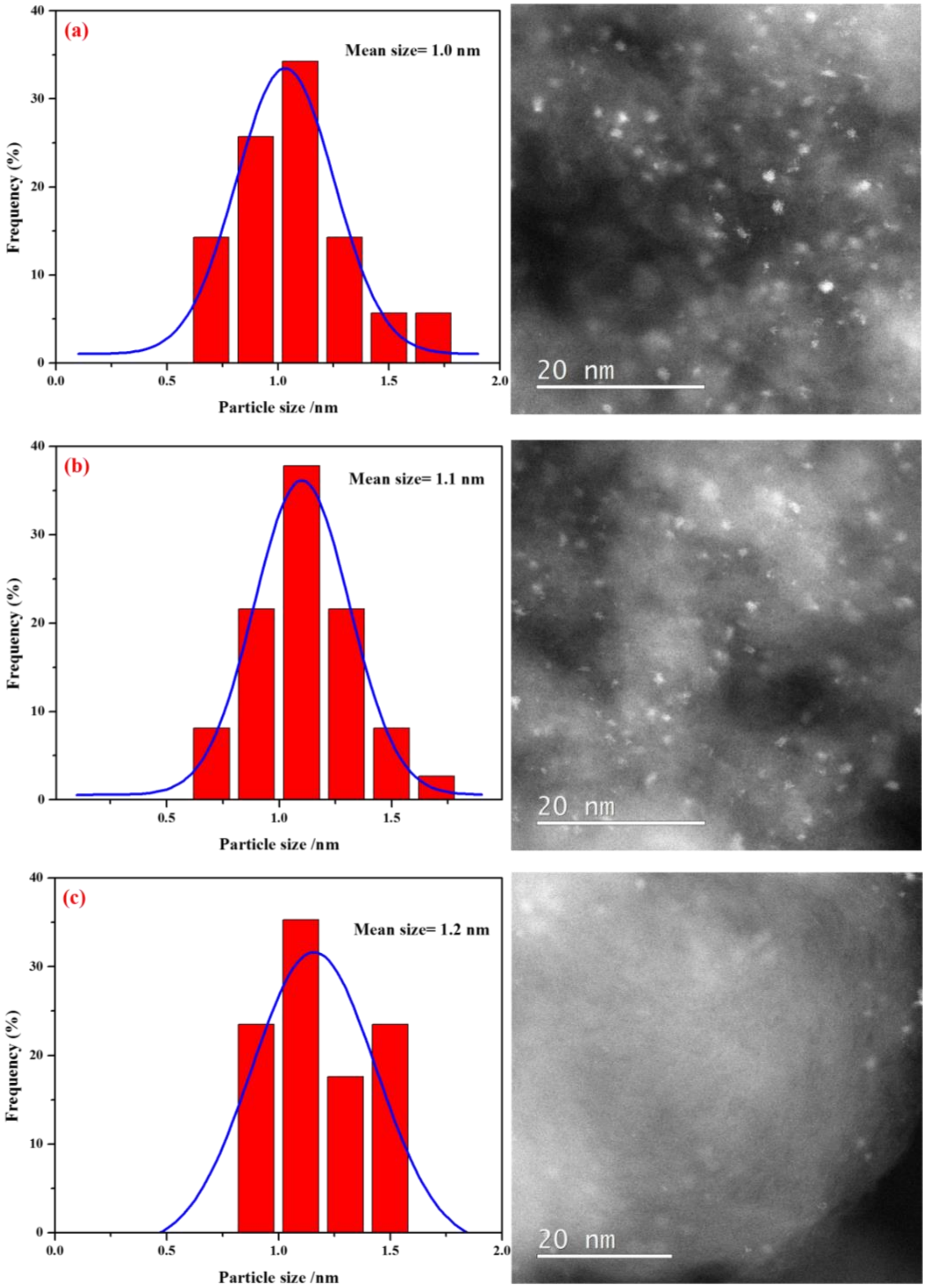

Figure S9 Pt particle size distributions and HAADF/STEM images of the used 0.25 wt.% Pt_1/XC-72, Related to Figure 1. The 0.25 wt.% Pt_1/XC-72 was used for 30 min at a reaction temperature of 40 ^{o}C.

As clearly shown in Figure S9 (a-c), the HAADF-STEM images confirm the sintering of the atomically dispersed 0.25 wt.% Pt_1/XC-72 into Pt clusters/particles during the hydrogenation reaction. The sintered Pt particles are uniformly distributed on the surfaces of the XC-72 carbon powders. Statistical analyses of the sintered Pt particles of the used 0.25 wt.% Pt_1/XC-72 catalyst yielded an average size of 1.1 ± 0.1 nm.

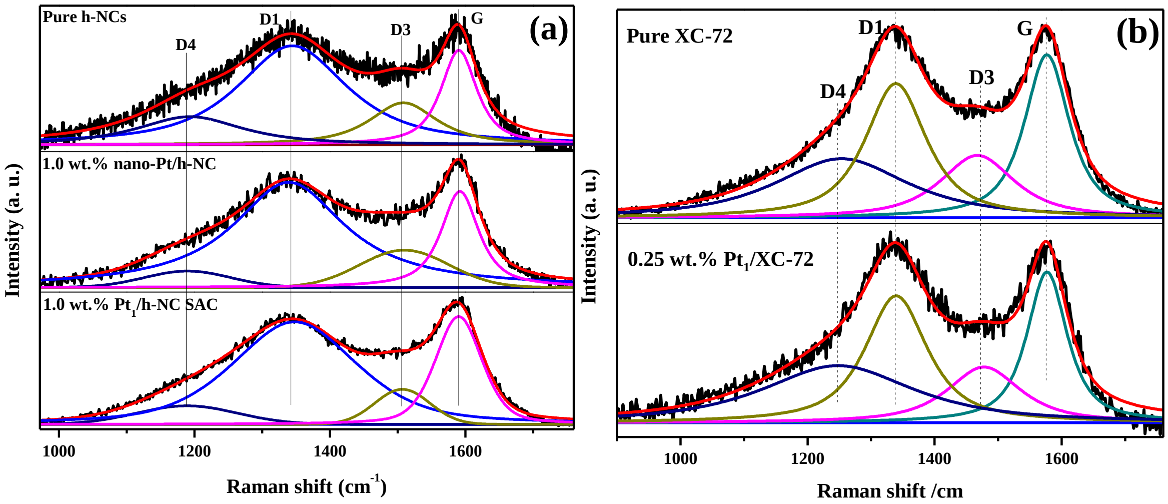

Figure S10 Raman spectra of carbon and carbon supported Pt catalysts, Related to Figure 2. Pure h-NCs, 1.0 wt.% nano-Pt/h-NC and 1.0 wt.% Pt_1/h-NC SAC (a); Pure XC-72 and 0.25 wt.% Pt_1/ XC-72 (b).

Raman spectroscopy, capable of providing useful information on the density of defects of carbon materials based on the ratio of D1 to G band intensities (I_{D1}/I_G) (Ferrari et al., 2000; Sadezky et al., 2005), was used to evaluate the carbon supports. As shown in Figure S10 (a-b), the peaks at 1190 cm^{-1}, 1345 cm^{-1}, and 1508 cm^{-1} can be assigned to the D4, D1 and D3 band and the peak at 1591 cm^{-1} can be assigned to the G band (Gopinadhan et al., 2013; Chernyak et al., 2017). The D_1-band is associated with the defects within the carbon atom plane and the G-band originates from the sp^2-bonded carbon atoms (Wang et al., 2016). The presence of the D3 band can be attributed to the presence of stacking faults, changes in the interlayer spacing, and the mixed breathing and asymmetric stretching vibrational modes of sp^2-carbons near defects (Maldonado et al., 2006; Smith et al., 2016). The broad component of D4 can be assigned to amorphous impurities in graphite materials (Prawer et al., 2000). The ratio between D1 and G band intensities (I_{D1}/I_G) can be used to quantify the degree of disorder and the presence of sp^3-hybridized carbon atoms within the structure. The pure h-NCs support exhibits a high I_{D1}/I_G ratio (1.1), suggesting the presence of high-density defects in the synthesized h-NCs (Wang et al., 2016; Wen et al., 2015). The ratio between D1 and G band intensities (I_{D1}/I_G) of pure XC-72 is 0.85, which is in line with the previously reported data (0.84) (Peng et al., 2017). The ratio between D1 and G band intensities (I_{D1}/I_G) of the 0.25 wt.% Pt_1/XC-72 is ~0.84.

The I_{D1}/I_G values for the h-NCs and XC-72 carbon are calculated to be 1.1 and 0.85 respectively, suggesting high density of edge/defect sites present in the h-NCs (Sadezky et al., 2005). From analyses of atomic resolution STEM images and Raman spectra we can confidently conclude that the synthesized h-NCs consist of numerous nanoscale graphene sheets with large number of edge sites. Prior to characterizing the carbon defects in the control carbon supports the pure carbon powders were pretreated/exposed to the identical condition that was used for synthesizing Pt single atom catalysts. Therefore, the effect of the chemical processes of adding the Pt single atoms onto the carbon supports on the Raman spectroscopy measurement was significantly minimized.

The experimentally measured I_{D1}/I_G ratio of the 1.0 wt.% Pt_1/h-NC SAC decreased from 1.1 to 0.9. We propose that the drop in the I_{D1}/I_G ratio is caused by the strong anchoring of Pt atoms to the edge/defect sites of graphene sheets, which are evidenced in the STEM images (Figure 1d and S8). For the XC-72 carbon, the I_{D1}/I_G ratio slightly decreased by ~1%, suggesting that the atomically dispersed Pt atoms were not strongly affect the XC-72 carbon, which corroborates STEM results that Pt atoms sintered to particles (~ 1.1 nm) after the hydrogenation reaction. Since the density of the Pt particles in the 1.0 wt.% nano-Pt/h-NC is extremely low, the I_{D1}/I_G ratio of 1.0 wt.% nano-Pt/h-NC is only decreased by 2%.

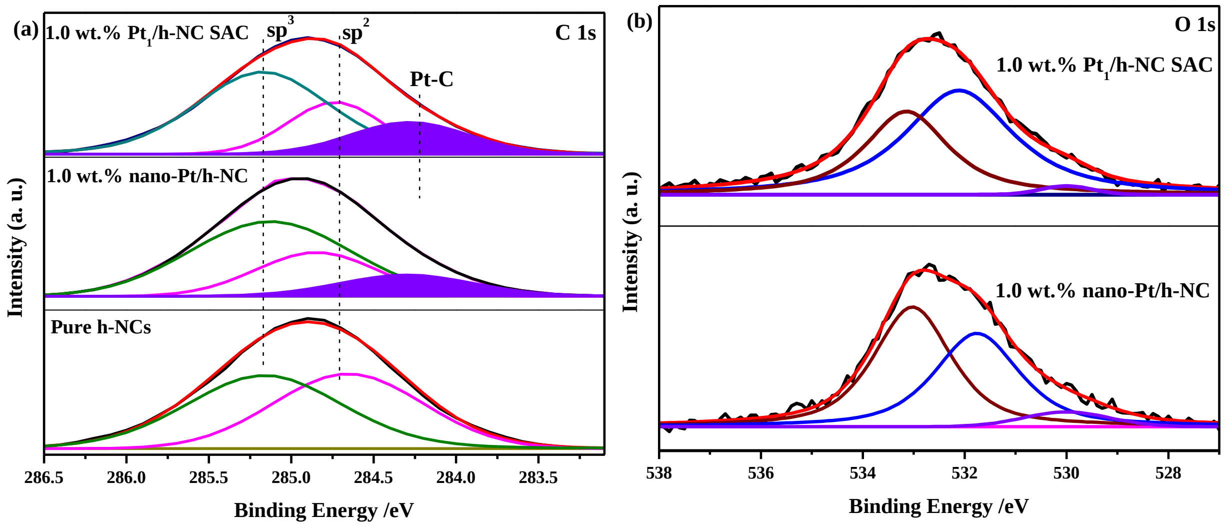

Figure S11 X-ray Photoelectron Spectroscopy of h-NCs and h-NC supported Pt catalysts: C 1s spectra (a) and O 1s (b), Related to Figure 2.

XPS spectra of the fresh 1.0 wt.% nano-Pt/h-NC catalyst and the pure h-NCs show only the carbon sp^2 (284.7 eV) and sp^3 (285.2 eV) peaks. However, the XPS spectrum, obtained from the 1.0 wt.% Pt_1/h-NC SAC, shows, in addition to the carbon sp^2 (284.7 eV) and sp^3 (285.2 eV) peaks, a new component located at 284.3 eV, assignable to a Pt-C bond originating from strong covalent interaction between Pt atoms and under-coordinated carbon atoms of the h-NCs (Rajasekaran et al., 2012; Ng et al., 2010). Our XPS data suggest that charge transfer occurred between the anchored Pt atoms and the edge carbon atoms in the h-NC mesoporous carbon. Such charge transfer results in oxidized Pt atoms, $Pt^{\delta+}$ ($0< \delta <2$), which can have catalytic properties different from those of the zero valent Pt^0. Similarly, the XPS C1s spectrum of the 1.0 wt.% nano-Pt/h-NC shows that in addition to the carbon sp^2 (284.7 eV) and sp^3 (285.2 eV) peaks, there is also a weak peak at 284.3 eV, probably originating from the presence of a small amount of Pt-C bond in the 1.0 wt.% nano-Pt/h-NC catalyst.

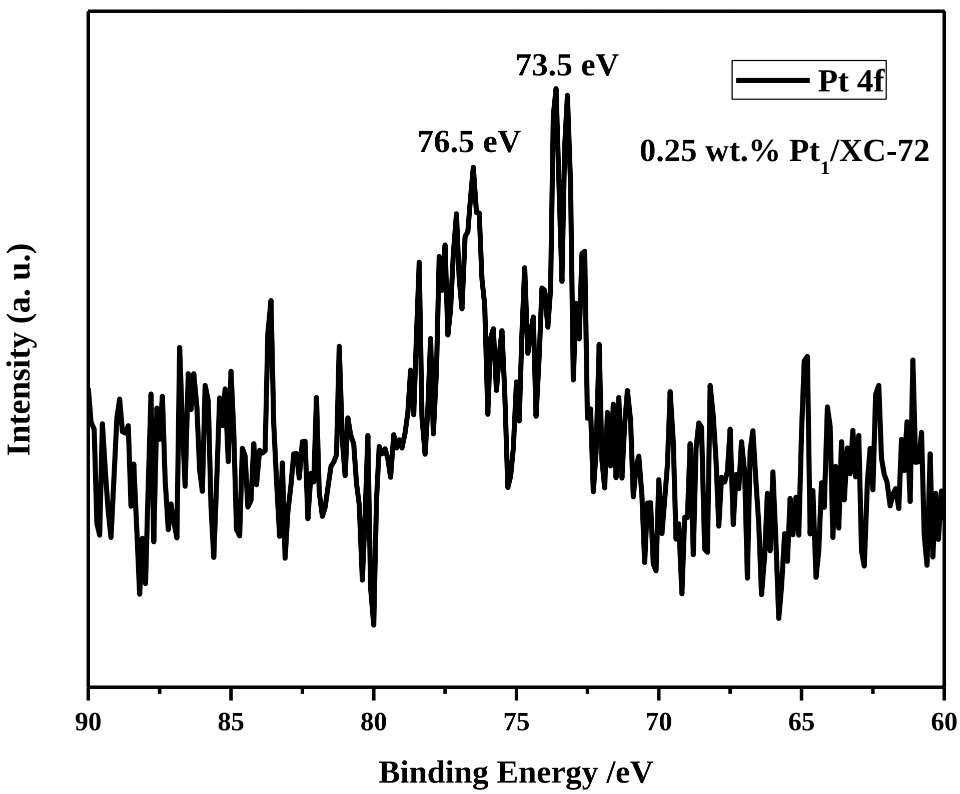

Figure S12 XPS Pt 4f spectrum of atomically dispersed 0.25 wt.% Pt_1/XC-72, Related to Figure 2.

The Pt 4f XPS spectrum of the 0.25 wt.% Pt_1/XC-72 catalyst could not be quantitatively analyzed due to the signal-to-noise problem (Ref 32 and 43). But the obvious peak located in the range of 73.2 and 73.5 eV as shown in Figure S12 indicates the oxidation state of Pt atoms in 0.25 wt.% Pt_1/XC-72 catalyst should be between +2 (72.6 eV) (Alderucci et al., 1995; Axnanda et al., 2015) and +4 (74.1 eV) (Peuckert et al., 1984; Ono et al., 2010).

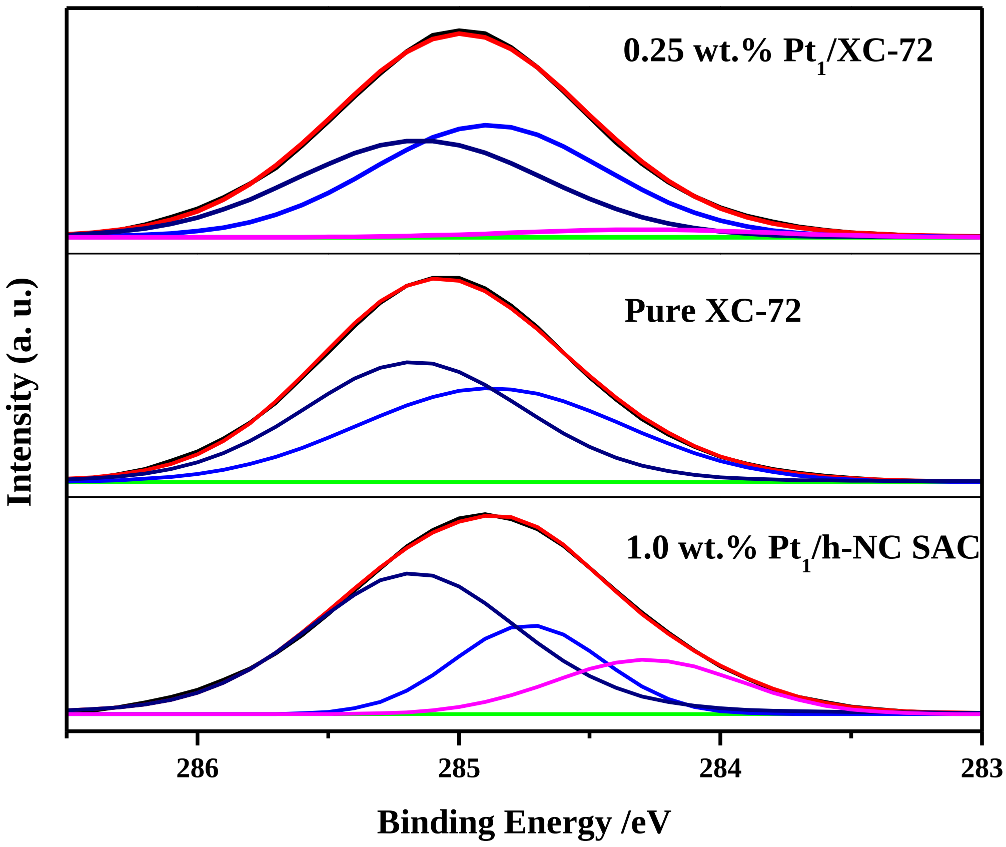

Figure S13 XPS C 1s spectra of: 1.0 wt.% Pt_1/h-NC SAC, atomically dispersed 0.25 wt.% Pt_1/XC-72 and pure XC-72, Related to Figure 2.

The C 1s XPS spectrum of the 0.25 wt.% Pt_1/XC-72 is similar to that of the pure XC-72 carbon, mainly displaying sp^2 (284.7 eV) and sp^3 (285.2 eV) components as shown in Figure S13. The absence of an obvious Pt-C peak in the 0.25 wt.% Pt_1/XC-72 suggests that the interaction of the atomically dispersed Pt atoms with the graphitic carbon support is much weaker than that between the Pt single atoms and the h-NCs in the 1.0 wt.% nano-Pt/h-NC SAC.

Table S1 Summary of the defect density and crystallite size of the synthesized Pt SACs and nano-Pt catalysts, Related to Table 1.

Sample	La /nm[a]	Defect density[b]	Defect density[c]
h-NCs	6.6 (1.2[d])	2.27E+12 (6.94E+13[e])	7.21E+11 (2.21E+13[e])
1.0 wt.% Pt_1/h-NC SAC	7.2	1.92E+12	6.11E+11
1.0 wt.% nano-Pt/h-NC	6.7	2.21E+12	7.04E+11
XC-72	14.2 (2.6[d])	4.96E+11 (1.48E+13[e])	1.58E+11 (4.71E+12[e])
1.0 wt.% Pt/XC-72	14.6	4.66E+11	1.48E+11

a: The crystallite size (La) is estimated based on the La (nm)=560*(El^4)*$(I_D/I_G)^{-1}$, El is the excitation energy laser energy used in the Raman experiment in eV units and the integrated intensities (peak areas) of the D and G bands instead of the ratio of peak amplitudes is used (Cançado et al., 2006). And the estimated value is normally larger than that of actual one (Chen et al., 2009).

b: The defect density (n_a/cm^{-2}) is calculated by the formula of $n_a=(1/La)^2$ (Cançado et al., 2006).

c: The defect density (n_a/cm^{-2}) is calculated by the formula of $n_a=10^{14}/\pi La^2$ (Zhong et al., 2014; Cancado et al., 2011).

d: The crystallite size is calculated based on the STEM images as shown in Figure S3 and Figure S6.

e: The defect density is calculated based on crystallite size in the parenthese.

Based on the estimate from the published two empirical formula models (Cançado et al., 2006; Zhong et al., 2014; Cancado et al., 2011), it is clear that the defect density of the synthesized h-NCs is ~5 times higher than that of the XC-72 carbon. After Pt deposition, the defect density of the 1.0 wt.% Pt_1/h-NC SAC is significantly decreased in comparison to pure h-NCs. Since the density of the Pt particles in the 1.0 wt.% nano-Pt/h-NC is extremely low, the defect density is only decreased by 4% due to Pt deposition, in comparison to pure h-NCs.

Transparent Methods

Preparation of single atom catalysts

Single Pt atoms were dispersed onto, via an adsorption method (Qiao et al., 2015; Lou et al., 2017; Lou et al., 2017), the surfaces of commercial carbon black (Vulcan XC-72) and home-made hollow nanocarbons (h-NCs). The precursors of Pt were hexachloroplatinic acid (Sigma-Aldrich).

1) Preparation of h-NCs. The h-NCs were synthesized via catalytic reforming/decomposition of ethanol on the surfaces of the ZnO nanowires. The details of synthesizing ZnO nanowires are discussed in the (Xu and Liu, 2016). In this work, we used the ZnO nanowires as templates to synthesize mesoporous h-NCs. The pre-formed ZnO nanowires were loaded into a high temperature furnace tube, which was then heated to 500 ^{o}C. A mixture of nitrogen, water and ethanol was then introduced into the furnace tube. Ethanol reforming as well as decomposition occurs on the surfaces of the ZnO nanowires. Such catalytic reactions result in residues of carbonaceous species uniformly coating the ZnO nanowires. Because of this coking induced deactivation, the thickness of the deposited carbonaceous layers is self-limiting. By controlling the reaction time, one can control the thickness of the deposited carbonaceous layers. After the desired thickness of the carbonaceous layers was obtained the ZnO (nanowires) were evaporated at a temperature of 750 ^{o}C for 6 h with a flowing gas mixture of H_2/Ar to produce the hollow carbon tubes with mesopores on the sidewalls.

2) Pt_1/h-NC preparation. The h-NCs were firstly dispersed in the ethanol and then the corresponding Pt precursors were slowly dropped into the ethanol. After being stirred at room temperature for 2 hours, the ethanol was slowly evaporated off and then the obtained precipitates were dried at 60 ^{o}C for 12 hours in air without further calcination. The actual Pt loading is 0.93 wt.% by ICP-MS.

3) Pt_1/XC-72 preparation. The carbon black powders (Vulcan XC-72) were firstly dispersed in the ethanol and then the corresponding Pt precursors were slowly dropped into the ethanol. After being stirred at room temperature for 2 hours, the ethanol was slowly evaporated off and then the obtained precipitates were dried at 60 ^{o}C for 12 hours in air. The actual Pt loading is 0.08 wt.% by ICP-MS.

4) Nano-Pt/h-NC preparation. The h-NC supported Pt nano particle catalyst (nano-Pt/h-NC) was prepared as control catalyst. The corresponding Pt precursors were firstly reduced by the $NaBH_4$ to form nano-meter sized Pt particles and then the above solution was slowly dropped into the ethanol to mix with the pre-synthesized h-NCs. After being stirred at room temperature for 2 hours, the ethanol was slowly evaporated off and then the precipitates were dried at 60 ^{o}C for 12 hours in air without further calcination. The actual Pt loading is 0.84 wt.% by ICP-MS.

Evaluation of the catalytic performance

The hydrogenation of 3-nitrostyrene was conducted in a Parr reactor. Before the activity test, the catalyst was first put into the reactor, to which 8 ml ethanol was added, and then the reactor was charged with 5 bar hydrogen and heated at 40 °C for 40 min to allow for the reduction treatment of the catalyst. After that, the 3-nitrostyrene substrate, internal standard O-xylene and 8 ml ethanol, were put into the reactor. Then, the reactor was flushed with 5 bar hydrogen for 11 times. After being sealed, the autoclave was charged with H_2 until 5 bar, then it was heated to 40 °C under stirring to initiate the reaction. The molecular ratio of Pt atoms to 3-nitrostyrene molecules is 0.078%. After reaction, the products were analyzed by gas chromatography (Agilent GC 7890A with HP-5 column) equipped with the Agilent auto sampler. The peaks were calibrated by the standard chemicals from Sigma-Aldrich. The turnover number (TON) value was measured by keeping the substrate conversion below 25% by tuning the molecular ratio of Pt atoms to 3-nitrostyrene molecules and the corresponding TON was calculated based on the total Pt loading in the catalyst, and the yields at the corresponding low conversion rate.

Catalyst Characterization

The loading level of the Pt was measured by a ThermoFinnegan iCAP Q quadrupole ICP-MS with CCT (Collision Cell Technology). Samples were run in KED (Kinetic Energy Discrimination) mode, with in-line aspiration of a multi-element internal standard.

Sub-angstrom resolution high-angle annular dark-field (HAADF) scanning transmission electron microscopy (STEM) images were obtained on a JEM-ARM200F TEM/STEM with a guaranteed resolution of 0.08 nm. Before microscopy examination, the catalyst powders were ultrasonically dispersed in ethanol and then a drop of the solution was put onto a copper TEM grid coated with a thin lacey carbon film.

The X-ray photoelectron spectroscopy (XPS) investigation was conducted on a Vacuum Generators 220i-XL using a mono-chromated Al Kα X-ray source (1486.6 eV). The samples were mounted onto the double-sided adhesive tape on the sample holder. The XPS spectra of the selected elements were measured with the constant analyzer pass energy of 20.0 eV. All binding energies (BEs) were referred to the C 1s peak (284.6 eV). The deconvolution protocol of the spectra is similar to our previous work (Lou et al., 2014; Lou et al., 2014). Raman experiments were conducted on LabRam HR 800 using 532 nm laser. The deconvolution protocol of the spectra was similar to that reported in literature (Wen et al., 2017).

The BET surface areas were measured by nitrogen adsorption at liquid nitrogen temperature by using a surface area and porosity analyzer (Quantachrome NOVA 4000e apparatus). Before measurement, the samples were degassed at 180 °C for 6 h in vacuum.

Supplemental References

Cancado, L. G., Jorio, A., Ferreira, E. H., Stavale, F., Achete, C. A., Capaz, R. B., Moutinho, M. V., Lombardo, A., Kulmala, T. S., and Ferrari, A. C. (2011). Quantifying defects in graphene via Raman spectroscopy at different excitation energies. Nano Lett. *11 (8)*, 3190-3196.

Chen, J. H., Cullen, W. G., Jang, C., Fuhrer, M. S., and Williams, E. D. (2009). Defect scattering in graphene. Phys. Rev. Lett. *102 (23)*, 236805.

Chernyak, S. A., Ivanov, A. S., Maslakov, K. I., Egorov, A. V., Shen, Z., Savilov, S. S., and Lunin, V. V. (2017). Oxidation, defunctionalization and catalyst life cycle of carbon nanotubes: a Raman spectroscopy view. Phys. Chem. Chem. Phys. *19 (3)*, 2276-2285.

Gopinadhan, K., Shin, Y. J., Yudhistira, I., Niu, J., and Yang, H. (2013). Giant magnetoresistance in single-layer graphene flakes with a gate-voltage-tunable weak antilocalization. Phys. Rev. B *88 (19)*, 195429.

Lou, Y., and Liu, J. (2017). A highly active Pt-Fe/gamma-Al_2O_3 catalyst for preferential oxidation of CO in excess of H_2 with a wide operation temperature window. Chem. Commun. *53*, 9020-9023.

Lou, Y., and Liu, J. (2017). CO Oxidation on Metal Oxide Supported Single Pt atoms: The Role of the Support. Ind. Eng. Chem. Res. *56*, 6916-6925.

Lou, Y., Cao, X. M., Lan, J., Wang, L., Dai, Q., Guo, Y., Ma, J., Zhao, Z., Guo, Y., Hu, P., and Lu, G. (2014). Ultralow-temperature CO oxidation on an In_2O_3-Co_3O_4 catalyst: a strategy to tune CO adsorption strength and oxygen activation simultaneously. Chem. Commun. *50*, 6835-6838.

Lou, Y., Ma, J., Cao, X., Wang, L., Dai, Q., Zhao, Z., Cai, Y., Zhan, W., Guo, Y., Hu, P., Lu, G., and Guo, Y. (2014). Promoting Effects of In_2O_3 on Co_3O_4 for CO Oxidation: Tuning O_2 Activation and CO Adsorption Strength Simultaneously. ACS Catal. *4*, 4143-4152.

Maldonado, S., Morin, S., and Stevenson, K. J. (2006). Structure, composition, and chemical reactivity of carbon nanotubes by selective nitrogen doping. Carbon, *44 (8)*, 1429-1437.

Ono, L. K., Yuan, B., Heinrich, H., and Cuenya, B. R. (2010). Formation and Thermal Stability of Platinum Oxides on Size-Selected Platinum Nanoparticles: Support Effects. J. Phys. Chem. C *114 (50)*, 22119-22133.

Peng, J., Chen, N., He, R., Wang, Z., Dai, S., and Jin, X. (2017). Electrochemically Driven Transformation of Amorphous Carbons to Crystalline Graphite Nanoflakes: A Facile and Mild Graphitization Method. Angew. Chem. Int. Ed. *56 (7)*, 1751-1755.

Peuckert, M., and Bonzel, H. P. (1984). Characterization of oxidized platinum surfaces by X-ray photoelectron spectroscopy. Surf. Sci. *145* (1), 239-259.

Prawer, S., Nugent, K. W., Jamieson, D. N., Orwa, J. O., Bursill, L. A., and Peng, J. L. (2000). The Raman spectrum of nanocrystalline diamond. Chem. Phys. Lett. *332* (1-2), 93-97.

Smith, M. W., Dallmeyer, I., Johnson, T. J., Brauer, C. S., McEwen, J. S., Espinal, J. F., and Garcia-Perez, M. (2016). Structural analysis of char by Raman spectroscopy: Improving band assignments through computational calculations from first principles. Carbon, *100*, 678-692.

Wang, L., Sofer, Z., Bousa, D., Sedmidubsky, D., Huber, S., Matejkova, S., Michalcova, A., and Pumera, M. (2016). Graphane Nanostripes. Angew. Chem. Int. Ed., *55 (45)*, 13965-13969.

Wen, G., Wang, B., Wang, C., Wang, J., Tian, Z., Schlogl, R., and Su, D. S. (2017) Hydrothermal Carbon Enriched with Oxygenated Groups from Biomass Glucose as an Efficient Carbocatalyst. Angew. Chem. Int. Ed. *56* (2), 600-604.

Wen, G., Wu, S., Li, B., Dai, C., and Su, D. S. (2015). Active sites and mechanisms for direct oxidation of benzene to phenol over carbon catalysts. Angew. Chem. Int. Ed. *54 (13)*, 4105-4109.

Xu, J., and Liu, J. (2016). Facet-Selective Epitaxial Growth of δ-Bi_2O_3 on ZnO Nanowires. Chem. Mater. *28*, 8141-8148.

Yan, X., Duan, P., Zhang, F., Li, H., Zhang, H., Zhao, M., Zhang, X., Xu, B., Pennycook, S. J., and Guo, J. (2019). Stable single-atom platinum catalyst trapped in carbon onion graphitic shells for improved chemoselective hydrogenation of nitroarenes. Carbon *143*, 378-384.

Zhang, X., Guo, J., Guan, P., Liu, C., Huang, H., Xue, F., Dong, X., Pennycook, S. J., and Chisholm, M. F. (2013). Catalytically active single-atom niobium in graphitic layers. Nat. Commun. *4*, 1924.

Zhong, J. H., Zhang, J., Jin, X., Liu, J. Y., Li, Q., Li, M. H., Cai, W., Wu, D. Y., Zhan, D., and Ren, B. (2014). Quantitative correlation between defect density and heterogeneous electron transfer rate of single layer graphene. J. Am. Chem. Soc. *136 (47)*, 16609-16617.
